# White Blood Cell Counts to High-Density Lipoprotein Cholesterol Ratio, as a Novel Predictor of Long-Term Adverse Outcomes in Patients After Percutaneous Coronary Intervention: A Retrospective Cohort Study

**DOI:** 10.3389/fcvm.2021.616896

**Published:** 2021-07-08

**Authors:** Ting-Ting Wu, Ying-Ying Zheng, Wen-Juan Xiu, Wan-Rong Wang, Yi-Li Xun, Yan-Yan Ma, Patigvl Kadir, Ying Pan, Yi-Tong Ma, Xiang Xie

**Affiliations:** ^1^Department of Cardiology, First Affiliated Hospital of Xinjiang Medical University, Urumqi, China; ^2^Department of Cardiology, First Affiliated Hospital of Zhengzhou University, Zhengzhou, China

**Keywords:** mortality, coronary artery disease, percutaneous coronary intervention, white blood cell, high-density lipoprotein cholesterol

## Abstract

**Background:** White blood cell (WBC) counts and high-density lipoprotein cholesterol (HDL-C) are widely available in clinical practice. However, the predictive value for cardiovascular disease (CVD) is uncertain. In the present study, we firstly assessed the prognostic value of WBC to HDL-C ratio (WHR) in patients with coronary artery disease (CAD) who underwent percutaneous coronary intervention (PCI).

**Methods:** Six thousand and fifty patients with CAD after PCI from a retrospective cohort study (identifier: ChiCTR-INR-16010153) were evaluated initially. Three hundred and seventy-one patients were excluded due to HDL cholesterol data not available, malignancy, dementia, psoriasis or eczema, systemic connective tissue disorders, multiple sclerosis, chronic liver disease, and chronic obstructive pulmonary disorder. Finally, 5,679 patients were included in the study. The primary outcome was long-term mortality. Secondary endpoints were mainly major adverse cardiovascular and cerebrovascular events (MACCEs), defined as a combination of stroke, cardiac death, stent thrombosis, recurrent myocardial infarction, and target vessel revascularization. The mean follow-up time of this study was 35.9 ± 22.5 months. We defined the best cutoff value of MHR according to the receiver operating curve (ROC), and then patients were divided into high and low WHR groups according to the cutoff value. We analyzed the data in both an acute coronary syndrome group (ACS) and a stable CAD subgroup, respectively.

**Results:** Overall, there were 293 cases of long-term mortality during the follow-up period. According to the cutoff value (WHR = 8.25), 1,901 ACS patients were divided into high WHR group (*n* = 724) and low WHR group (*n* = 1,177). Compared to low WHR group, the incidence of all-cause mortality (ACM, 5.5 vs. 3.6%, *p* = 0.048) and cardiac death (4.7vs. 2.9%, *p* = 0.042) were significantly higher in the high WHR group. In stable CAD group, we also found the incidence of ACM and cardiac death were significantly higher in the high group compared to that in the low group. We did not find significant difference between the high and the low WHR group in the incidence of MACCEs. The multivariate Cox proportional hazards model showed that increased WHR level was independently correlated with the mortality. In the high WHR group, the risk of ACM increased two times in ACS [adjusted *HR* = 2.036 (1.258–3.296), *p* = 0.004] and 1.5 times in stable CAD [adjusted *HR* = 1.586 (1.178–2.136), *p* = 0.002].

**Conclusion:** The present study indicated that an increased WBC count to HDL-C ratio was independently associated with long-term mortality in CAD patients who underwent PCI.

## Introduction

An epidemic of cardiovascular diseases (CVD) in China is emerging, as a result of lifestyle changes, urbanization, and the accelerated process of aging. The incidence of CVD may continuously increase and will present an upward trend in the next decade. It has been reported that it will still be the leading cause of death in China, with two in five deaths attributed to CVD. Cardiovascular disease will account for 44.60 and 42.51% of all deaths in rural and urban areas, respectively (1). With the advances in medical technology, the application of percutaneous coronary stent implantation (PCI) in patients with coronary artery disease (CAD) is increasing (2). Therefore, identification of novel predictive markers for CAD is of more interest. It is known that inflammation plays a key role in atherosclerosis (AS). An increasing number of studies had concluded that the humoral biomarkers of inflammation are associated with the initiation, progression, and instability of atherosclerotic plaques and appear to be associated with future CVD events (3, 4). The total white blood cell (WBC) count is considered to be one of the simplest and most commonly measured markers of the immune response and inflammation. Many studies have reported that an elevated WBC is associated not only with higher CVD events incidence (5, 6) but also with the poor prognosis of CVD (7–10). Previous studies have shown that high-density lipoprotein cholesterol (HDL-C) could inhibit leukocytes, leading to the inhibition of activated leukocytes adhesion, proliferation, and control of the proliferation of progenitor cells differentiated into leukocytes, exerting anti-inflammatory and antioxidant effects (11, 12). Some observational studies have suggested a simplistic hypothesis that with a wide range of concentrations, HDL-C is an inverse predictor of CAD and mortality. A modest increase in HDL-C may even reduce cardiovascular risk to some extent (13–15). However, the above conclusions are still controversial. A recent study found that, on a continuous scale, the relationship between HDL-C and all-cause mortality (ACM) was U-shaped, both low and high concentrations of HDL-C were associated with high ACM (16). Some randomized clinical trials also have concluded that drug interventions that increase HDL-C levels can fail to reduce poor cardiovascular outcomes but can even exacerbate adverse events (17, 18). In view of the above evidence, the WBC to HDL-C ratio (WHR) could show a patient's inflammatory status. Using WHR, rather than using them each as a single indicator, may be more reliable and practical in predicting the occurrence, development, and prognosis of CVD. In this study, we investigate the predictive value of WHR for the clinical outcomes of CAD patients who have undergone PCI.

## Methods and Materials

**Figure 1:**
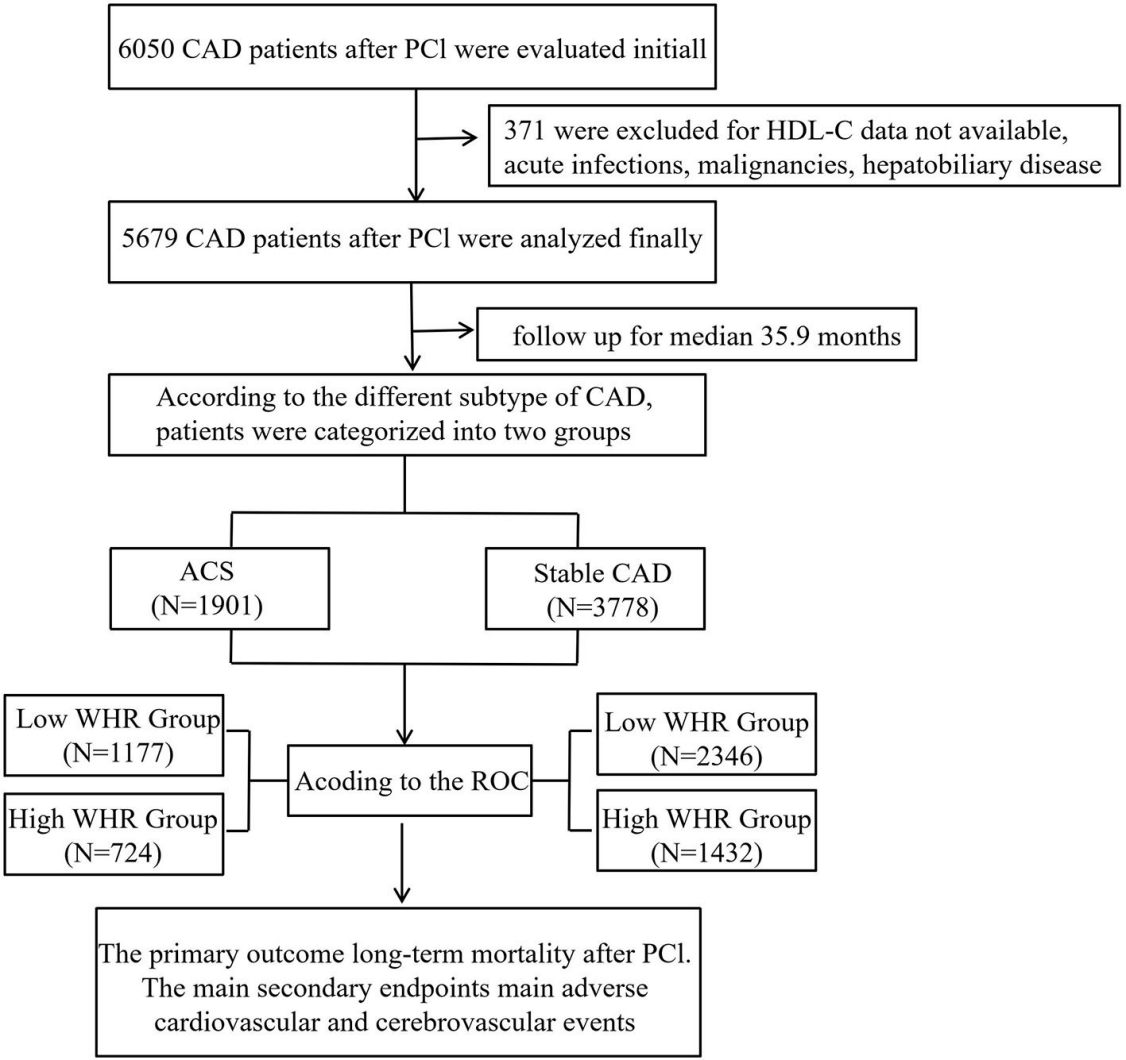
The follow chart of participants inclusion.

From January 2008 to December 2016, based on case records and a follow-up registry performed in the First Affiliated Hospital of Xinjiang Medical University, 6,050 CAD patients who underwent PCI were included and registered as “The Clinical Outcomes and Risk Factors of Patients with Coronary Heart Disease after PCI (CORFCHD-PCI) study.” The CORFCHD-PCI study was aimed at evaluating the clinical outcomes and risk factors of PCI patients. More details of the design can be found on http://www.chictr.org.cn (Identifier: ChiCTR-ORC-16010153) (19). By examine the medical records and/or telephone interviews of patients or their families to get the short- or long-term prognosis. It was approved by the ethics committee of the First Affiliated Hospital of Xinjiang Medical University. A total of 6,050 patients with CAD after PCI were evaluated initially. But 371 patients were excluded due to the lack of available HDL-C data, malignancy, dementia, psoriasis or eczema, systemic connective tissue disorders, multiple sclerosis, chronic liver disease, chronic obstructive pulmonary disorder, and alcohol problems. Finally, 5,679 patients were enrolled in the present study. The flow chart of inclusion and exclusion of participants is shown in Figure 1.

The definitions of hypertension, diabetes mellitus, and hypercholesterolemia have been described in detail elsewhere (19). Briefly, hypertension was considered as blood pressure ≥140 and/or ≥90 mm/Hg in at least three measurements or use of any anti-hypertensive drug. Diabetes mellitus is defined as fasting plasma glucose levels >126 mg/dl or current use of anti-diabetic medications. Hypercholesterolemia is considered as total serum cholesterol of >200 mg/dl or the use of lipid-lowering medication. Smoking and drinking status was collected from medical records. The smoking and drinking status of the patients was recorded in detail, including daily usage amount, years of smoking/drinking, and the duration of quitting. We finally defined their smoking and drinking status by recording and presenting it in binary fashion. The primary outcome was long-term mortality. The main secondary endpoints were major adverse cardiovascular and cerebrovascular events (MACCEs). End of follow-up for the main study for each participant was recorded as the date of the primary or secondary endpoint event that has been defined or the date of end of follow-up for the assessment center attended, whichever comes first (19). Demographic characteristics, echocardiography, and laboratory data were recorded, and all variables were measured in absolute quantities per unit volume using automated, clinically validated coulters on fresh samples. The use of blockers, angiotensin converting enzyme inhibitors (ACEI), angiotensin receptor blockers (ARB), statins, aspirin, clopidogrel, and calcium channel blockers (CCB) was recorded during the follow-up period.

All analyses were performed using SPSS 22.0 (SPSS Inc., Chicago, IL, USA). Continuous variables of normal distribution are expressed as mean ± standard deviation, and categorical variables are expressed as quantity and percentage. In order to compare parameter continuous variables, Student's *t*-test was used. In order to compare non-parametric continuous variables, the Mann-Whitney U-test was used. The chi-square test was used for the comparison of categorical variables. Results were reported in the form of hazard ratios (HRs) and 95% confidence intervals. A multivariate Cox model was used to determine the independent parameters of long-term mortality and MACCEs. The Kaplan-Meier method was used to construct the cumulative survival curves of primary results and secondary endpoints, and the log-rank test was used for comparison. A value of *p* < 0.05 was considered significant (19).

## Results

According to the different types of CAD, patients were divided into acute coronary syndrome (ACS) group and stable CAD group. The best cutoff value (WHR = 8.25) was found according to the receiver operating curve (ROC), and then patients were divided into high and low WHR groups. A number of variables were significantly different between the patients in the WHR groups. In ACS, participants with higher WHR were generally male, more likely younger, smoking, and with higher fasting blood glucose (FBG), higher triglycerides (TG), and lower HDL-C. In addition to having the same features as those found in ACS patients with high WHR, there were some additional features in the high WHR group of stable CAD as shown in Table 1.

**Table 1:** Clinical and laboratory characteristics.

**Variables**	**ACS (*****N*** **=** **1,901)**			**Stable CAD (*****N*** **=** **3,778)**		
	**Low group**	**High group**	***P***	**Low group**	**High group**	***P***
	**(*N* = 1,177)**	**(*N* = 724)**		**(*N* = 2,346)**	**(*N* = 1,432)**	
Age(years)	60.9 ± 10.7	58.1 ± 11.0	<0.0001	60.3 ± 10.4	57.6 ± 10.9	<0.001
Male sex [*n* (%)]	840 (71.4%)	569 (78.6%)	<0.0001	1676 (71.4%)	1138 (79.5%)	<0.001
Smoking [*n* (%)]	456 (38.7%)	327 (45.2%)	0.006	881 (36.3%)	646 (45.1%)	<0.001
Drinking [*n* (%)]	333 (28.3%)	227 (31.4%)	0.162	618 (26.3%)	488 (34.1%)	<0.001
EH [*n* (%)]	495 (42.1%)	322 (44.5%)	<0.316	990 (42.2%)	618 (43.2%)	0.564
Diabetes [*n* (%)]	277 (23.5%)	178 (24.6%)	0.619	561 (23.9%)	371 (25.9%)	0.173
BUN (mmol/l)	5.49 ± 1.74	5.56 ± 1.73	0.375	5.49 ± 1.58	5.56 ± 1.73	0.241
UA (mmol/l)	321.1 ± 90.9	327.2 ± 91.3	0.153	317.8 ± 86.8	331.7 ± 94.2	<0.001
Cr (mmol/l)	75.94 ± 21.70	77.85 ± 20.14	0.055	74.29 ± 18.79	77.79 ± 21.83	<0.001
FBG (mmol/l)	6.51 ± 3.01	6.89 ± 3.28	0.010	6.43 ± 3.06	6.83 ± 3.26	<0.001
TG (mmol/l)	1.76 ± 1.09	2.18 ± 1.53	<0.0001	1.77 ± 1.18	2.09 ± 1.36	<0.001
TC (mmol/l)	4.06 ± 1.10	3.79 ± 1.04	<0.0001	4.08 ± 1.14	3.77 ± 1.06	<0.001
HDL-C (mmol/l)	1.14 ± 0.51	0.81 ± 0.20	<0.0001	1.15 ± 0.58	0.81 ± 0.19	<0.001
LDL-C (mmol/l)	2.52 ± 0.92	2.30 ± 0.85	<0.0001	2.55 ± 0.95	2.35 ± 0.87	<0.001
SBP (mmHg)	127 ± 19	127 ± 19	0.999	127 ± 19	127 ± 19	0.695
DBP (mmHg)	76 ± 11	77 ± 12	0.092	76 ± 11	77 ± 12	0.210

*BUN, blood urea nitrogen; Cr, creatinine; UA, uric acid; FBG, fasting blood glucose; TG, triglyceride; TC, total cholesterol; HDL-C, high-density lipoprotein cholesterol; LDL-C, low-density lipoprotein cholesterol; SBP, systolic blood pressure; DBP, diastolic blood pressure; EH, essential hypertension*.

As shown in Table 2, after a mean follow-up period of 35.9 ±22.5 months, the incidence of long-term mortality was more frequent in the high WHR group. There were 293 cases of long-term mortality during the follow-up period. Compared with the low WHR group, the incidence of ACM (5.5 vs. 3.6%, *p* = 0.048) and cardiac death (4.7 vs. 2.9%, *p* = 0.042) were significantly higher in the high WHR group. In the stable CAD group, we also found the incidence of ACM and cardiac death were significantly higher in the high group compared with that in the low group. We did not find significant differences between the high and the low WHR group in the incidence of MACCEs.

**Table 2:** Clinical outcomes comparison between groups.

**Clinical outcomes**	**ACS (*****N*** **=** **1,901)**			**Stable CAD (*****N*** **=** **3,778)**		
	**Low group**	**High group**	***P***	**Low group**	**High group**	***P***
	**(*N* = 1,177)**	**(*N* = 724)**		**(*N* = 2,346)**	**(*N* = 1,432)**	
**Primary end point**						
Long-term mortality, *n* (%)	42 (3.6%)	40 (5.5%)	0.048	116 (4.9%)	95 (6.6%)	0.034
**Secondary endpoints**						
MACCEs, *n* (%)	160 (13.6%)	112 (15.5%)	0.281	328 (14.0%)	208 (14.5%)	0.665
MACEs, *n* (%)	147 (12.5%)	101 (14.0%)	0.363	296 (12.6%)	193 (13.5%)	0.454
Cardiac death, *n* (%)	34 (2.9%)	34 (4.7%)	0.042	116 (4.9%)	95 (6.6%)	0.009

*MACCEs, major adverse cardiovascular and cerebrovascular events; MACEs, major adverse cardiovascular events*.

After adjusting for age, sex, diabetes, hypertension, Cr, UA, FBG, TG, TC, HDL-C, and LDL-C, the multivariate Cox model showed that increased WHR level was independently correlated with long-term mortality. In the high WHR group, the incidence of mortality would be increased two times in ACS [adjusted *HR* = 2.036 (1.258–3.296), *p* = 0.004, Table 3], and 1.5 times in stable CAD patients [adjusted *HR* = 1.586 (1.178–2.136), *p* = 0.002, Table 4]. The risk of cardiac death increased significantly in the high WHR group of ACS (adjusted *HR* = 2.305 [1.360–3.907], *p* = 0.002).

**Table 3:** Cox regression analysis results for ACS long-term mortality.

**Variables**	***B***	***SE***	***X^**2**^***	***P value***	***HR (95%CI)***
Age	0.030	0.011	7.096	0.008	1.031 (1.008–1.054)
Sex	0.035	0.300	0.014	0.907	1.036 (0.575–1.864)
Smoking	0.047	0.289	0.027	0.870	1.048 (0.595–1.847)
Drinking	0.088	0.295	0.089	0.766	1.092 (0.613–1.946)
Diabetes	0.094	0.273	0.118	0.731	1.098 (0.644–1.874)
Hypertension	0.264	0.235	1.266	0.260	1.303 (0.822–2.065)
Cr	0.007	0.005	2.175	0.140	1.007 (0.998–1.016)
UA	0.001	0.001	1.301	0.254	1.001 (0.999–1.004)
FBG	0.006	0.038	0.027	0.868	1.006 (0.934–1.085)
TG	−0.053	0.095	0.310	0.578	0.948 (0.787–1.143)
TC	−0.083	0.166	0.249	0.618	0.921 (0.665–1.274)
HDL-C	0.164	0.180	0.826	0.363	1.178 (0.828–1.675)
LDL-C	0.071	0.191	0.139	0.709	1.074 (0.739–1.561)
WHR high vs. low	0.711	0.246	8.384	0.004	2.036 (1.258–3.296)

*Cr, creatinine; UA, uric acid; FBG, fasting blood glucose; TG, triglyceride; TC, total cholesterol; HDL-C, high-density lipoprotein cholesterol; LDL-C, low-density lipoprotein cholesterol; WHR, white blood cell counts to high-density lipoprotein cholesterol ratio*.

**Table 4:** Cox regression analysis results for stable CAD long-term mortality.

**Variables**	***B***	***SE***	***X^**2**^***	***P-value***	***HR (95%CI)***
Age	0.031	0.007	19.376	<0.001	1.032 (1.017–1.046)
Sex	−0.012	0.178	0.004	0.948	0.988 (0.697–1.402)
Smoking	−0.046	0.181	0.063	0.802	0.955 (0.669–1.364)
Drinking	−0.112	0.195	0.328	0.567	0.894 (0.610–1.310)
Diabetes	0.021	0.179	0.014	0.906	1.021 (0.719 -1.450)
Hypertension	0.174	0.146	1.421	0.233	1.191 (0.894–1.586)
Cr	0.004	0.003	1.240	0.265	1.004 (0.997–1.010)
UA	0.000	0.001	0.332	0.565	1.000 (0.998–1.001)
FBG	−0.011	0.024	0.214	0.643	0.989 (0.943–1.037)
TG	−0.004	0.055	0.004	0.949	0.996 (0.895–1.110)
TC	−0.223	0.103	4.727	0.030	1.250 (1.022–1.529)
HDL	0.122	0.119	1.041	0.308	1.129 (0.894–1.426)
LDL	−0.287	0.124	5.359	0.021	0.750 (0.588–0.957)
WHR high vs. low	0.461	0.152	9.213	0.002	1.586 (1.178–2.136)

*Cr, creatinine; UA, uric acid; FBG, fasting blood glucose; TG, triglyceride; TC, total cholesterol; HDL-C, high-density lipoprotein cholesterol; LDL-C, low-density lipoprotein cholesterol; WHR, white blood cell counts to high-density lipoprotein cholesterol ratio*.

**Figure 2:**
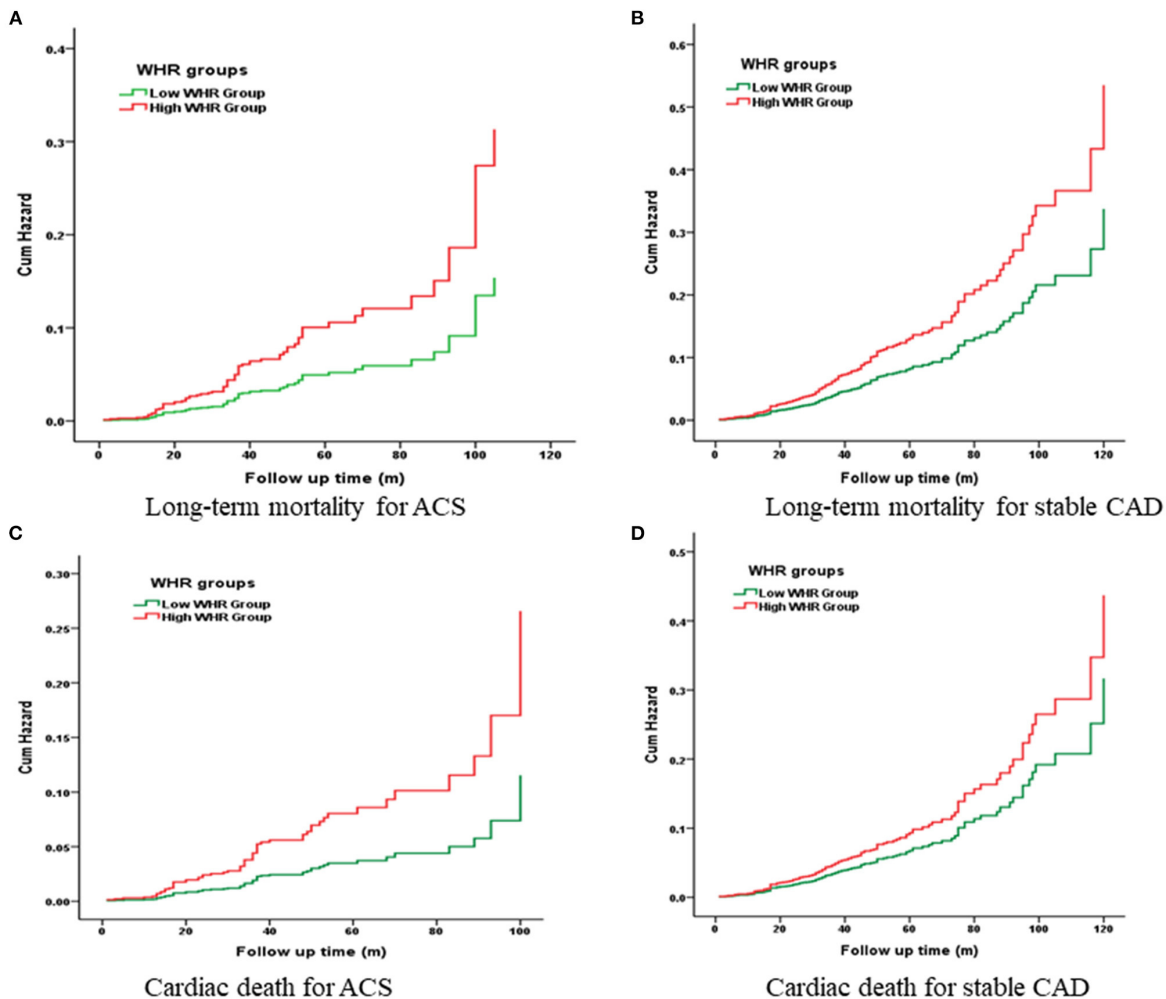
Cumulative Kaplan-Meier estimates of the time to the first adjudicated occurrence of primary endpoint and secondary endpoints.

Kaplan-Meier curves showed a significant gradually increased risk in the high WHR group, which represents more long-term mortality and cardiac death rates as WHR increased (Figure 2).

## Discussion

In our present study, we found that a higher WHR was associated with a significant increased risk of long-term mortality and a 2.3-fold increased risk of cardiac death for ACS. To our knowledge, this is the first time WHR has been used as a novel biomarker to assess the prognostic value of PCI patients. The statistical power and reliability were improved by a large sample size and a long-term follow-up of 10 years.

It is acknowledged that the underlying cause of AS was of inflammatory origin. While biomarkers of inflammation are numerous, leukocyte counts have attracted attention as a cheap test, and studies have consistently shown that leukocyte counts are associated with an increased risk of CVD events. White blood cell counts were found to be independently associated with impaired myocardial reperfusion in acute ST segment elevation myocardial infarction (STEMI) patients with PCI (20). Karahan et al. (21) found higher WBC at admission was an independent predictor of impaired microvascular perfusion in patients with STEMI. Acute myocardial infarction treated with PCI with elevated WBC had poor 6-month left ventricular functional recovery (22). Research about the relationship between WBC and the mortality of CVD have also been reported. Similar to previous findings (23–28), our study also found that WBC was associated with long-term outcomes in CAD patients. Elevated WBC at baseline could be an independent risk factor for long-term mortality in CAD patients receiving PCI. However, it has shown a limited predictive power in our study [adjusted *HR* = 1.123 (1.075–1.172), *p* < 0.001]. The ratio of WBC to HDL-C has been proposed as a potentially better predictor of adverse outcome risk than total WBC alone. Moreover, we found the predictive value of WHR in stable CAD and ACS was different. Despite the development of new therapeutic methods, ACS is the most hazardous type of CAD. Unstable atherosclerotic plaques activated by systemic inflammation is a typical characteristic of ACS (29). Although a large number of clinical markers have been used for risk stratification, most of them are still far from clinical application. Our present study suggested that WHR was significantly related to the cardiac death of ACS but not stable CHD. This may be due to the possible consequence of severe systemic inflammatory and neuroendocrine responses by leukocytes being more serious in ACS. Therefore, we believe the WHR may have a role in the clinical management of ACS patients because of its easy and wide application.

What mechanisms might explain the association of WHR with CAD mortality? The inflammatory and dyslipidemia hypothesis of CAD has been well-established. Currently, there is evidence that inflammation plays a significant role in the occurrence and progression of AS, and an active inflammatory process may trigger plaque rupture, increase the risk of coronary thrombosis, and lead to clinical ischemic events (30). Decades of accumulated experimental and clinical evidence have brought inflammation to the forefront as a common mechanism, linking traditional and emerging risk factors with changes in arterial wall cell behavior and the accumulation and activation of WBCs in arterial lesions (31). Cellular atherosclerotic plaques appear more prone to rupture. It is assumed that leukocytes may play a direct role in destabilizing the plaque itself by thinning the fibrous cap (26). High-density lipoprotein cholesterol is a group of lipoprotein particles with a density >1.063 g/ml and a size between 7 and 12 nm (32). High-density lipoprotein cholesterol particles consist of approximately 30% phospholipids, 25% cholesterol (70% of which is esterified), and 5% TG (33). Protein is the main structure and function composition of HDL-C. ApoA-I, which makes up 70% of the composition of total HDL-C, was thought to be the major structure and function protein of HDL-C, in interaction with cell receptors, and to give HDL-C multiple resistance activity of AS (34). The concept of HDL-C as a beneficial type is still widely accepted, as is its anti-atherosclerosis function. Reverse cholesterol transport (RCT), removing cholesterol from the surrounding tissues (macrophage foam cells) and then transporting it to the liver, where it is excreted as bile and feces, which prevents cholesterol accumulation in macrophages and blood vessels and prevents the formation of AS (35). HDL-C could prevent or reverse endothelial dysfunction through the following mechanisms. Play a direct endothelial protection function by promoting the production of vasoactive molecules (such as Nitric oxide, NO) and down-regulates cell adhesion molecules level (36); Play anti-inflammatory and anti-oxidant effects by inducing decreased expression of vascular cell adhesionmolecule-1 (VCAM-1), intercellular adhesionmolecule-1 (ICAM-1) and E-selectin, by inhibiting activation of nuclear factor kB(NF-kB) and its downstream expression; Affect the proliferation of smooth muscle cells by inducing the decreased expression of cyclin D1, NF-κB and cyclocytose-2 (37, 38). Most of the functional properties of HDL-C do not depend on the cholesterol content of the particles themselves but on the many other structural components contained in the complex and highly heterogeneous HDL-C particles. High-density lipoprotein cholesterol that can have normal functions is called “functional HDL,” while in the acute stage, chronic inflammation, and some metabolic diseases, HDL-C will undergo a series of pathological modifications, leading to changes in its components and functional groups, thus leading to the concept of “dysfunctional HDL-C” (38). Cholesterol metabolism in macrophages is an important factor to maintain cholesterol homeostasis *in vivo* and a necessary way to eliminate cholesterol in the body. Macrophages are the key cells affecting the occurrence and development of AS. The main characteristic of AS is that oxidized low-density lipoprotein (OXLDL) invades the subcutaneous layer and is engulfed by macrophages to form foam cells, which then develop into atherosclerotic plaques. The outflow of cholesterol from the lipid-loaded macrophages in the vascular wall can prevent the occurrence of AS. Cholesterol transport in macrophages is a complex process regulated by multiple genes, including ATP-binding cassette transporter A1, ATP binding cassette transporter G1, neutral CE hydrolase, apoA-I, apoE, lecithin cholesterol acyl transferase, cholesteryl ester transfer protein, low-density lipoprotein receptor, CD36, and scavenger receptor class B type I (39, 40). The factors above exert different degrees of regulatory roles alone or in combination in regulating macrophage cholesterol transport. Therefore, these molecules can be used as targets for clinical treatment of AS, but their exact roles in human physiology and disease processes still need to be further studied. Most of the previous studies have shown that HDL-C molecules could inhibit the migration of macrophages and the oxidation of low-density lipoprotein molecules, and they could promote the efflux of cholesterol from these cells to exert anti-inflammatory and antioxidant effects (41, 42); further studies on HDL-C metabolism and its relationship with macrophage cholesterol transport pathway proteins are expected to provide some new therapeutic targets for inhibiting and reversing human AS lesions. Above all, WHR can be seen as a pro-inflammatory and pro-oxidative factor in these processes. It could be a novel predictor of long-term adverse outcomes in patients after PCI.

## Study Limitations

Nonetheless, there were some limitations to this study. The first limitation was the retrospective design of the study and the single-center nature of the study. Second, additional inflammation markers, such as C-reactive protein, pro-inflammatory cytokines, angiotensin II and norepinephrine levels, etc., were not evaluated. Third, we used a single blood sample during the pre-procedural period, which might be insufficient to predict the persistence of the WHR ratio over time. Therefore, the result should be verified by a multi-center, prospective study to confirm the association between increased WHR and adverse outcomes.

## Conclusion

The increased WHR ratio independently increased the long-term mortality in CAD patients after PCI, and it could be a novel biomarker for poor prognosis.

## Data Availability Statement

Due to confidentiality policies, data will not be shared. Please contact the corresponding author for any inquiries.

## Ethics Statement

The ethics committee or review committee of the First Affiliated Hospital of Xinjiang Medical University approved the research protocol. Because the study was a retrospective cohort study based on real-world situations, there was no need to obtain informed consent from patients.

## Author Contributions

T-TW, Y-YZ, and W-JX made substantial contributions to the study conception and design and to the drafting and critical revision of the manuscript for important intellectual content. W-RW, Y-LX, Y-YM, PK, and YP made substantial contributions to the study conception and design and to the critical revision of the manuscript for important intellectual content. XX and Y-TM made substantial contributions to the study conception and design and to the drafting and critical revision of the manuscript for important intellectual content, including study supervision. The authors read and approved the final manuscript.

## Conflict of Interest

The authors declare that the research was conducted in the absence of any commercial or financial relationships that could be construed as a potential conflict of interest.
